# Supplementary material for: Content-rich biological network constructed by mining PubMed abstracts
Source: BMC Bioinformatics. 2004 Oct 8;5:147. doi: 10.1186/1471-2105-5-147 (PMC528731; doi:10.1186/1471-2105-5-147)
Supplement: Additional File 2 — The original results of the above study (non-essential files are deleted to keep the file size under the limit set by BMC bioinformatics). [file 1471-2105-5-147-S2.bz2 › chilibotAdditionalFile2/dip05/719ID11777003E18056/html/RPOE_RSEA.html]

 


 **RPOE** and **RSEA** 
  
Found 4 abstracts in PubMed, retrieved 4.  
 

 What does Google say? 
 PDF only 
| .edu only 

---

**Interactive relationship** (e.g. stimulation, inhibition, etc)

**Neutral relationship**- Elevation of active sigma E levels in Escherichia coli by either repressing the expression of  **rseA**  encoding an anti sigma E factor or cloning  **rpoE**  in a multicopy plasmid, led to a large decrease in the number of dead cells and the accumulation of cellular proteins in the medium in the stationary phase.  Ref: 10960110 J Bacteriol, 2000

**Non-interactive relationship** (e.g. studied together, co-existance, homology, etc.)

- Modulation of the Escherichia coli sigmaE  **RpoE**  heat shock transcription factor activity by the  **RseA** , RseB and RseC proteins.  Ref: 9159522 Mol Microbiol, 1997
- The expression of htrA is regulated by a complex set of signal transduction pathways, which includes an alternative sigma factor,  **RpoE** , an anti sigma factor,  **RseA** , a two component regulatory system, CpxRA, and two phosphoprotein phosphatases, PrpA and PrpB.  Ref: 9383148 Mol Microbiol, 1997
